# Human papillomavirus (HPV) vaccination program in Sri Lanka: Ongoing costs and operational context of a routinized program

**DOI:** 10.1016/j.jvacx.2024.100456

**Published:** 2024-02-08

**Authors:** Frédéric Debellut, Deepa Gamage, Sandeep Kumar, Sashimali Wickramasinghe, Thilanga Ruwanpathirana, Manjula Kariyawasam, Chinthana Sanjeewa Perera, Samitha Ginige, Nirmala Cooray, Clint Pecenka, Rose Slavkovsky, D. Scott LaMontagne, Mercy Mvundura

**Affiliations:** a Center for Vaccine Innovation and Access, PATH, Geneva, Switzerland; b Epidemiology Unit, Ministry of Health, Colombo, Sri Lanka; c PATH, New Delhi, India; d Center for Vaccine Innovation and Access, PATH, Seattle, USA; e John Snow, Inc., Arlington, USA^1^; f Product and Market Advancement, PATH, Seattle, USA

**Keywords:** HPV vaccine, Immunization delivery cost, Sri Lanka, Costing

## Abstract

Existing evidence on the cost of human papillomavirus (HPV) vaccination programs has focused on pilot and demonstration projects or initial introductions, which resulted in a perceived high cost. We aimed to study the ongoing cost and operational context of an established HPV vaccination program in Sri Lanka.

We conducted a retrospective operational research and microcosting study focusing on 2019. We collected data from 30 divisional health units, 10 districts, and the central level. We then evaluated financial and economic costs, reported by level of the health system, program activity, cost types, and per dose delivered.

In 2019, Sri Lanka delivered a total of 314,815 doses of HPV vaccine. In our study sample, 95 % of the HPV vaccination sessions took place at schools, with peaks of delivery in February–March and September–October. The weighted mean financial cost per dose delivered was $0.27 (95 % confidence interval [CI]: $0.15–$0.39) and the economic cost per dose was $3.88 (95 % CI: $2.67–$5.10), excluding the cost of vaccines and supplies. Most of the cost was borne by the divisional health unit level. Service delivery and social mobilization were major contributors to overall costs at the divisional health unit level, and vaccine collection or distribution and storage were the most costly activities at the district and central levels. Cost drivers included the opportunity cost of health worker and non-health worker time at the divisional health unit level and capital costs for vehicles and equipment, along with fuel, maintenance, and energy, at the district and central levels.

This study provides new evidence on the cost and cost drivers of a routinized HPV vaccination program. Results can be used for financial planning purposes in Sri Lanka and may inform other countries as they consider use of HPV vaccines.

## Introduction

Nearly all cases of cervical cancer can be attributed to infection from oncogenic human papillomavirus (HPV). It remains the fourth most common cancer in women globally, with an estimated 340,000 deaths worldwide in 2020. Low- and middle-income countries (LMICs) are disproportionately affected, with about 90 % of new cases and deaths occurring in these settings [1]. In Sri Lanka, the cervical cancer crude incidence rate is estimated at 9.9 per 100,000 women according to the National Cancer Control Programme, and the age-standardized mortality rate is 4.9 per 100,000 women [2].

HPV vaccines have been available since 2006, and the World Health Organization (WHO) recommends their use as a primary prevention strategy against cervical cancer [3]. As of June 2023, 132 countries have introduced HPV vaccines in their national immunization program, mostly in high-income countries [4], [5]. Underlying reasons for slower adoption in LMICs include, but are not limited to, concerns over the affordability of the vaccine, global supply constraints in recent years, and the perceived higher cost of delivery related to reaching a target population outside the routine infant immunization program [6], [7]. HPV vaccination programs may be less able to achieve the cost efficiencies that occur with vaccines targeting infants, where service delivery costs can be shared [8].

Existing research provides evidence that the cost to deliver HPV vaccines to adolescent girls is generally higher than for vaccines targeting infants. However, this evidence is mostly focused on costs of demonstration projects where HPV vaccines were introduced in small geographic areas or during the introduction of HPV vaccines [9], [10], [11], [12], [13], [14], [15]. Evidence for the programmatic and economic costs of delivering HPV vaccine in national programs that have been operating for years is sparse, limiting our understanding of financial needs to maintain HPV vaccinations in a national Expanded Programme on Immunization (EPI) schedule.

Sri Lanka first introduced HPV vaccine in September 2017, targeting girls in Grade 6 (10 years of age) [4]. Sri Lanka is a lower-middle-income country which graduated from Gavi, the vaccine alliance, in 2015. Nevertheless, the country received exceptional financial support from Gavi for the introduction of HPV vaccine. Sri Lanka also has access to Gavi pricing for HPV vaccine and the government of Sri Lanka fully finances vaccine procurement. HPV vaccine is implemented through the National Immunization Programme as a routine vaccination of two doses delivered with a minimum of six months between doses. HPV vaccine delivery is mainly school-based and is part of the country’s school health program. Sri Lanka reported 82 % coverage of the second dose of HPV vaccine in 2019 [16].

As the HPV vaccination program in Sri Lanka has been in place for since 2017, it provides an opportunity to understand the routine or ongoing cost to operate a country-wide, school-based program. To improve understanding of ongoing HPV vaccination program costs, we conducted a cross-sectional evaluation of the ongoing costs and operational context of the two-dose HPV vaccination schedule program in Sri Lanka, focusing on costs to the health system.

## Methods

### Study objectives and design

This study collected data on cost and operational context of the HPV vaccination program in Sri Lanka. We focused on calendar year 2019 as our study reference year, to minimize recall bias while preventing any interference due to the COVID-19 pandemic, and to capture an entire pre-pandemic annual period during which the HPV vaccination program operated. However, circumstances related to the COVID-19 pandemic’s response and to the economic crisis that followed prevented us to collect primary data until June 2022. Cost data were collected from the health system perspective. No data were collected on the cost to households from seeking HPV vaccination.

In line with costing principles recommended by available guidelines for costing of immunization programs, we used a microcosting, ingredients-based approach to collect data on a number of identified program activities and cost categories [17]. We evaluated the following program activities: vaccine procurement; estimating demand; program planning and management; social mobilization and IEC (information, education, and communication); routine training; vaccine collection or distribution and storage; service delivery; supervision; record keeping; waste management; and crisis management. Each activity and their related cost components which we evaluated are available in supplementary Table 1. For each of these activities, we evaluated both financial and economic costs. Financial costs represent direct expenses or monetary outlays incurred during the program implementation. Economic costs represent a broader perspective, accounting for the opportunity cost of resources that would have otherwise been allocated to other activities and programs, in addition to financial costs [17]. We did not include the cost of vaccines or injection supplies in our analysis. Cost categories included in financial costs were, for example, per diems, meeting costs, fuel for vehicles, energy for cold chain equipment, and shipping, handling, and customs for vaccines. The cost categories included in the opportunity costs were the time for health workers and non-health workers and the annualized costs for vehicles and equipment.

To understand the program operational context, we used implementation science methods by adapting questions from the WHO HPV Post-Introduction Evaluation (PIE) tool to understand what was done, how, and how often, to deliver HPV vaccines over the study period [18].

### Study area, sampling, and data collection

The Sri Lankan health system is geographically organized within 9 provinces, 26 districts, and 357 divisional health units, the latter representing the level for health care delivery. Within each divisional health unit, schools falling under the jurisdiction area are involved in the school health program. The school health program is a combined program for health promotion, prevention (including immunization), and medical checkups and screening. HPV vaccination is provided by public health inspectors, nursing officers, and midwives through the school health program, in coordination with the epidemiology unit at the central level and directors and medical officers for health at the district level [19].

Primary data collection took place in five purposively selected provinces to account for geographic and cultural differences, including the Central, Eastern, Northern, Southern, and Western provinces. In these five provinces, random sampling was performed using the Sampling Design Optimizer to select 10 districts and 30 divisional health units [20].

Two structured questionnaires were developed and administered at the national level and in the selected districts and divisional health units. At the central level, respondents were members of the epidemiology unit, responsible for immunization activities in the country. At the district level, the immunization focal point and responsible person for the vaccine store were the main respondents. Finally, at the divisional health unit, the focal person for providing the HPV immunization services was the main respondent. Data capture was done electronically using the Open Data Kit application. We also extracted data on vaccination sessions at each divisional health unit. These data were tabulated to calculate total doses delivered and categorize vaccine delivery strategies. Additional secondary data collection obtained staff salaries, replacement prices for equipment, prices for fuel and energy, and vaccine stock data for the year 2019. The supplementary Table 2 contains secondary data used in the analysis. Cost data analysis was done using Stata (version 17.0), while Excel, SPSS, and SAS were used to analyze the operational research data. Different members of the study team conducted the operational data analysis, using similar methods but leveraging analytical software they had.

### Cost considerations

All cost data were collected in Sri Lankan rupees (LKR) and converted to US$ using the 2019 exchange rate of LKR 178.74 for US$1 [21]. For equipment, we annualized capital costs over the useful life year of each equipment type, using replacement prices and applying a discount rate of 3 % [22].

For resources that are shared with the broader immunization program, we performed cost allocations. For example, we used the proportion of the number of HPV vaccine doses delivered among all vaccine doses delivered by the program to allocate immunization staff time spent on collecting and transporting HPV vaccines. We used the proportion of the volume (cm^3^) of HPV vaccines delivered among the total volume of all vaccines delivered to allocate the cost of capital equipment, fuel and maintenance costs for vehicles, and energy and maintenance costs for cold chain equipment to the HPV vaccination program (supplementary Table 2). We also relied on respondents reporting the time spent on HPV vaccination as part of other immunization activities they undertake, and valued the associated cost using salaries corresponding to each staff position.

Using weights originating from the sampling, we calculate and report weighted average financial and economic costs for each program activity, per cost category and per level of the health system, as well as total weighted financial and economic program costs and per dose delivered.

### Ethical considerations

The PATH Research Determination Committee reviewed this study and determined it did not involve human subjects nor represent research. In Sri Lanka, the study protocol received ethical approval from the National Hospital of Sri Lanka’s Ethics Review Committee (AAJ/ETH/COM/2021/NOVEMBER).

## Results

### Context of the HPV vaccination program in Sri Lanka

All program activities that took place in 2019 for the HPV vaccination program in Sri Lanka are described in Table 1. Program planning and management was conducted by all divisional health units in the sample as well as 9 out of 10 districts and at the national level. Social mobilization activities were conducted mostly at the divisional health unit level with 76 % of facilities carrying out mostly sensitization meetings involving health workers, school staff, and other local stakeholders. A smaller proportion of facilities organized trainings of health workers (17 out 30 facilities). Four districts organized training sessions. Vaccines were mostly delivered to divisional health units and districts from the higher level. Only two divisional health units reported having to collect vaccines from the vaccine store on occasion. Vaccine distribution is entirely executed using vehicles owned by the Ministry of Health. Almost all divisional health units (90 %) reported receiving supervision visits from either the district or national level, and most of these visits were solely focused on HPV vaccine. All facilities ensure record-keeping activities, either by maintaining paper-based reporting forms or using an electronic reporting system. Only 5 out of 30 facilities in the sample reported having responded to a crisis event during the period of analysis, and most activities conducted were in response to fertility or vaccine safety concerns. Waste management is organized monthly, with waste collected and disposed of at the nearest hospitals.

**Table 1:** HPV vaccine delivery key program characteristics at different levels of the health system in 2019.

	**Divisional health unit level** **(n = 30)**	**District level** **(n = 10)**	**National level** **(n = 1)**
**Vaccine procurement**	60 % of divisional health units ordered HPV vaccines monthly while others ordered less frequently.	70 % of districts ordered HPV vaccines monthly.	Three HPV vaccine shipments were received for a total of 325,000 doses.
**Estimating demand**	All divisional health units reported their target population.Mean target population at divisional health unit level was 550 girls (min 61–max 588) .	All districts reported their target population.Mean target population at district level was 7,272 girls (min 1,875–max 17,000) .	National level reported a target population of 173,130 girls.
**Program planning and management**	All divisional health units reported doing planning and management activities with an average of 14 activities reported.	9 of the 10 study districts completed program planning and management activities.	The national level organized two program management events, including one involving lower levels.
**Social mobilization and IEC (information, education, and communication)**	23 (76 %) divisional health units carried out social mobilization and IEC activities. The majority (83 %) of activities were sensitization meetings, done by 21 divisional health units, and involving health workers, school staff, and other local stakeholders.	One district organized sensitization meetings for schools. One district organized distribution of IEC materials.	No social mobilization or IEC activity reported at the national level.
**Training**	19 (63 %) divisional health units reported no ongoing/routine training for HPV vaccination. Of the 22 activities done by the remaining divisional health units, the most common (81 %) training reported was for health workers.	Four districts organized HPV vaccination trainings.	No training organized at national level.
**Vaccine collection or distribution and storage**	29 (97 %) divisional health units have HPV vaccine delivered to them by the district level. When divisional health units made trips to collect vaccine, a vehicle owned by the facility was used.	All districts had vaccines delivered from the national level. Four districts also collected vaccines on occasion.	The national level delivered vaccines to district stores, and vaccines and supplies were always delivered together.
**Service delivery**	80 % of divisional health units reported HPV vaccines were delivered with other interventions, including other vaccines, health education, deworming, vitamin A, and school medical inspection. Average sessions per facility was 25.6. Average HPV vaccine doses per facility was 761.	Mean doses delivered was 11,354.	Total doses delivered was 314,815.
**Supervision**	27 (90 %) facilities reported receiving supervision visits, and most were solely for HPV vaccines.	Six districts reported supervision trips to lower levels. The number of trips ranged between 1 and 11.	The national level organized 21 supervision trips in 2019.
**Record keeping**	19 (63 %) facilities reported maintaining and tallying their own data then reporting to the district level; the rest used an electronic reporting system.	All districts reported either using an electronic reporting system or using record-keeping materials from previous years. Two districts reported making copies of record-keeping materials.	Not applicable.
**Waste management**	Waste was collected monthly and disposed of at the nearest hospitals.	Not applicable.	Not applicable.
**Crisis management**	5 (17 %) facilities reported responding to a crisis event. Most activities were in response to fertility or safety concerns with HPV vaccines.	No crisis management activity at the district level.	No crisis management activity at the national level.

**Fig. 1:**
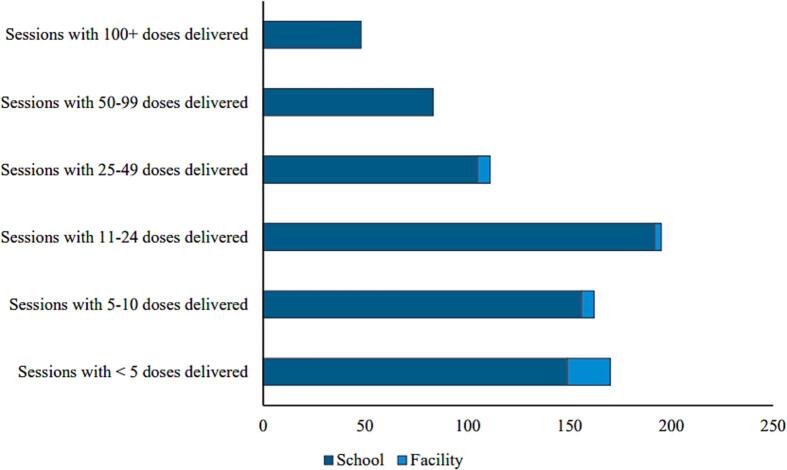
Total HPV vaccination sessions per size and location in 2019. Abbreviation: HPV, human papillomavirus.

**Fig. 2:**
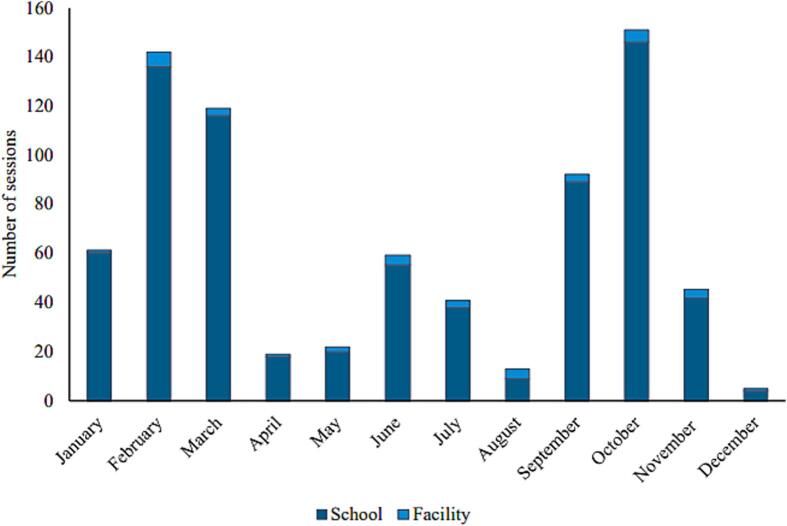
HPV vaccination sessions by month and location. Abbreviation: HPV, human papillomavirus.

During 2019, the 30 divisional health units in the sample delivered a total of 22,821 doses of HPV vaccine via 769 vaccination sessions. Almost all sessions organized by divisional health units in our sample (95 %) were conducted at schools, and the rest were conducted at the divisional health units. No outreach sessions or vaccination occurred in the community through the HPV immunization program. The majority of vaccination sessions delivered fewer than 25 doses (Fig. 1), and sessions happened throughout the year with two peaks in February–March and September–October (Fig. 2), corresponding to both the dosing schedule and the start/end of the school year. HPV vaccines were delivered to the target population with most doses given to girls aged 10 and 11 years old, corresponding to the age of attendance of junior secondary education Grade 6.

### Cost of the HPV vaccination program in Sri Lanka at the divisional health unit level

At the divisional health unit level, the weighted mean annual financial cost for the HPV vaccination program was $189 (95 % confidence interval [CI]: $124–$255). The majority (97 %) of the financial cost was for service delivery activities (Table 2). The weighted mean annual economic cost was $3,190 (95 % CI: $2,019–$4,360). The economic cost was mostly composed of costs for service delivery (71 %) and social mobilization and IEC (15 %). Financial costs represented a small share, less than 6 %, of economic costs.

**Table 2:** Weighted mean financial and economic costs of HPV vaccination program activities at divisional health units (n = 30) in 2019 US$.

	**Financial costs**	**Economic costs**
**Mean costs by program activity and percent of total costs**		
Service delivery	$183 (97 %)	$2,457 (71 %)
Estimating demand	$0 (0 %)	$15 (<1%)
Vaccine procurement	$0 (0 %)	$64 (2 %)
Program planning and management	$0 (0 %)	$54 (2 %)
Social mobilization and IEC	$0 (0 %)	$463 (15 %)
Training	$0 (0 %)	$18 (<1%)
Crisis management	$0 (0 %)	$16 (<1%)
Vaccine collection or distribution and storage	$5 (3 %)	$39 (1 %)
Waste management	$0 (0 %)	$0 (0 %)
Record keeping	$0 (0 %)	$64 (2 %)
Supervision	n/a	n/a
**Total costs** (weighted mean and 95 % CI)	**$189** **[$124–$255]**	**$3,190** **[$2,019–$4,360]**
**Cost per dose** (weighted mean and 95 % CI)	**$0.22** **[$0.13–$0.31]**	**$3.70** **[$2.56–$4.85]**

Abbreviations: CI, confidence interval; HPV, human papillomavirus; IEC, information, education, and communication.

**Fig. 3:**
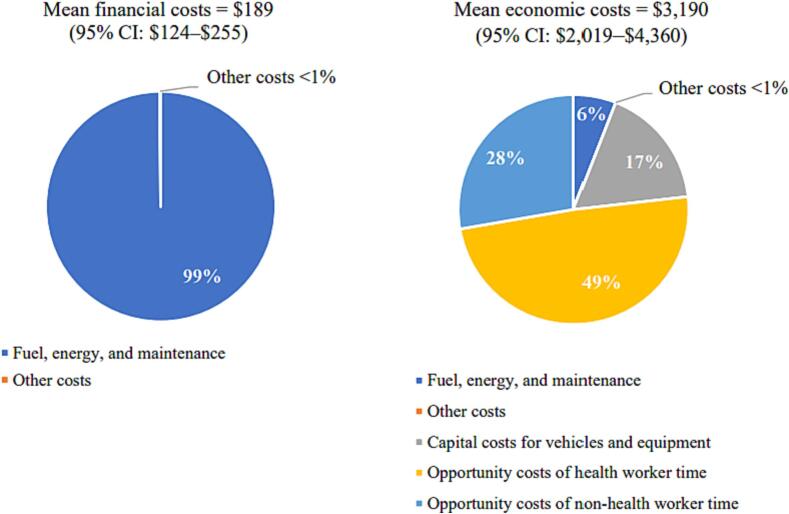
Financial and economic cost types at the divisional health unit level. Abbreviations: CI, confidence interval.

At the divisional health unit level, the main driver of financial cost was fuel, energy, and maintenance for vehicles and the cold chain (Fig. 3). The largest driver of economic cost was the opportunity cost of health worker time, representing almost half the economic cost (49 %), while opportunity cost of non-health worker time and capital costs for vehicles and equipment represented 28 % and 17 %, respectively, of the economic cost (Fig. 3). Fuel, energy, and maintenance represented only 6 % of the economic cost.

With a total of 22,821 doses provided at the divisional health unit level during the year, the financial cost per dose was $0.22 (95 % CI: $0.13–$0.31) and the economic cost per dose was $3.70 (95 % CI: $2.56–$4.85).

### Cost of the HPV vaccination program in Sri Lanka at the administrative levels

At the district level, the weighted mean annual financial cost was $459 (95 % CI: $190–$729) and the weighted mean annual economic cost was $1,807 (95 % CI: $843–$2,770). Financial cost represented 25 % of the economic cost (Table 3). Vaccine collection or distribution and storage was the main program activity, contributing 96 % of financial costs. This was also the main contributor to economic costs, representing 78 % of the total economic cost. Human resources represented 21 % of the economic cost.

**Table 3:** Weighted mean financial and economic costs of HPV vaccination program activities at the administrative levels in 2019 US$.

**Costs by program activity**	**District level (n = 10)**		**National level (n = 1)**	
	**Financial costs**	**Economic costs**	**Financial costs**	**Economic costs**
Estimating demand	$0 (0 %)	$0 (0 %)	$0 (0 %)	$0 (0 %)
Vaccine procurement	$0 (0 %)	$0 (0 %)	$443 (28 %)	$443 (6 %)
Program planning and management	$16 (3 %)	$16 (<1%)	$0 (0 %)	$0 (0 %)
Social mobilization and IEC	$0 (0 %)	$3 (<1%)	$0 (0 %)	$0 (0 %)
Training	$0 (0 %)	$0 (0 %)	$0 (0 %)	$0 (0 %)
Crisis management	$0 (0 %)	$0 (0 %)	$0 (0 %)	$0 (0 %)
Vaccine collection or distribution and storage	$442 (96 %)	$1,416 (78 %)	$1,121 (72 %)	$5,499 (72 %)
Waste management	$0 (0 %)	$0 (0 %)	$0 (0 %)	$0 (0 %)
Record keeping	$1 (<1%)	$1 (<1%)	$0 (0 %)	$0 (0 %)
Supervision	$0 (0 %)	$0 (0 %)	$0 (0 %)	$0 (0 %)
Human resources	$0 (0 %)	$371 (21 %)	$0 (0 %)	$1,714 (22 %)
**Total costs** (weighted mean and 95 % CI)	**$459** ($190–$729)	**$1,807** ($843–$2,770)	**$1,564**	**$7,657**
**Cost per dose** (weighted mean and 95 % CI)	**$0.04** ($0.01–$0.07)	**$0.16** ($0.09–$0.23)	**$0.01**	**$0.02**

Abbreviations: CI, confidence interval; HPV, human papillomavirus; IEC, information, education, and communication.

At district and national levels, some program activities reported zero costs even though activities took place, due to how data collection for vehicle use and health worker time was done at these levels.

**Fig. 4:**
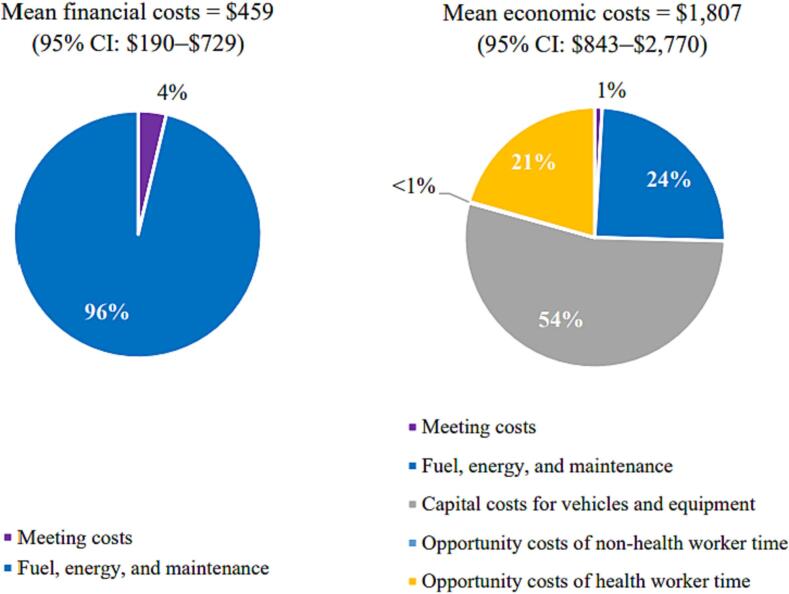
Financial and economic cost types at the district level. Abbreviations: CI, confidence interval.

The main cost driver at the district level was fuel, maintenance, and energy, representing 96 % of financial costs (Fig. 4). These represented only 24 % of the economic costs at the district level. The main driver of economic costs at this level was the capital cost of vehicles and equipment, representing 54 %. The opportunity cost of health worker time represented 21 % of the total economic costs.

With a mean number of 11,354 HPV vaccine doses delivered by the 10 districts in our sample in 2019, the financial cost per dose was $0.04 (95 % CI: $0.01–$0.07) and the economic cost per dose was $0.16 (95 % CI: $0.09–$0.23), as shown in Table 3.

**Fig. 5:**
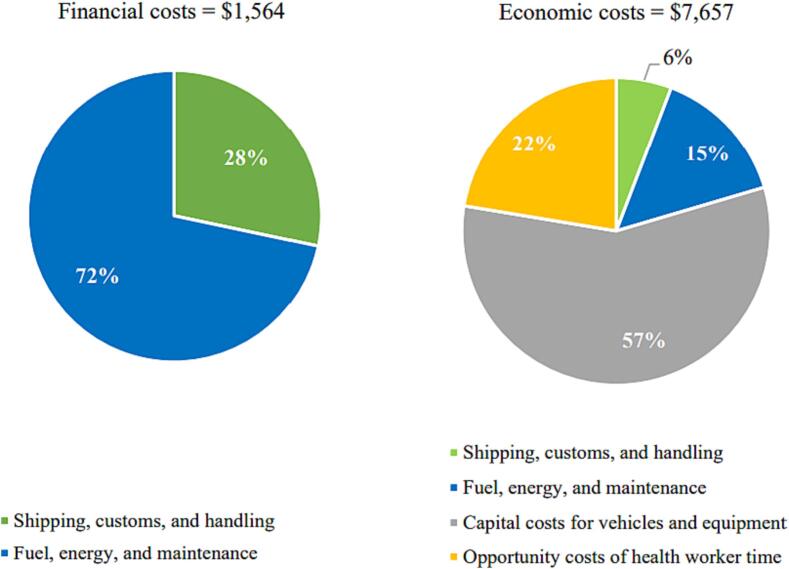
Financial and economic cost types at the national level.

At the national level, the total financial cost of the HPV vaccination program in 2019 was $1,564 (Table 3). The total economic cost was $7,657. Financial costs represented about 20 % of the economic costs. Similar to the district level, the main program activity contributing to the financial cost at national level was vaccine collection or distribution and storage, representing 72 % of the financial cost. The other activity contributing significantly to financial cost was vaccine procurement, representing 28 % of the total. Fuel, energy, and maintenance were the main cost driver of financial costs (72 %) at national level (Fig. 5). The other cost driver was shipping, customs, and handling charges, representing 28 % of the financial costs. For the national level, the capital cost of vehicles and equipment was the main driver of economic costs, representing 57 % of the total. Other cost types of relevance included the opportunity cost of health worker time and fuel, energy, and maintenance, representing 22 % and 15 %, respectively, of the total economic cost at the national level.

With a total of 314,815 doses of HPV vaccine delivered in 2019 in the entire country, the financial cost per dose at the national level was $0.01 and the economic cost per dose was $0.02.

### Aggregated cost of the HPV vaccination program in Sri Lanka

**Fig. 6:**
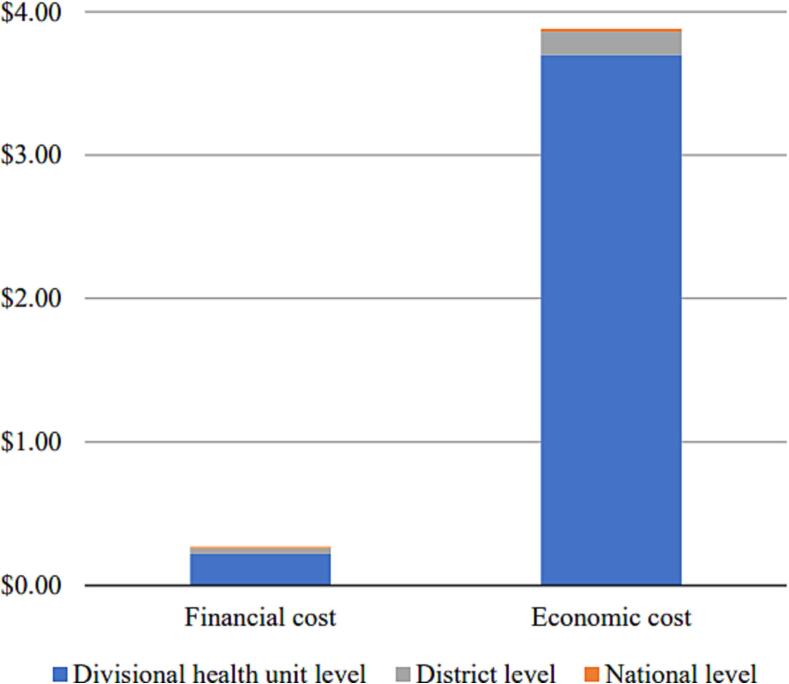
Mean financial and economic costs per dose aggregated across all levels of the health system.

The aggregated financial cost per dose (all levels together) was $0.27 (95 % CI: $0.15–$0.39) and the aggregated economic cost per dose was $3.88 (95 % CI: $2.67–$5.10). The divisional health unit level bore the greatest share of the cost per dose (Fig. 6). Service delivery represented 73 % of the aggregated economic cost, the second activity contributing to economic cost was social mobilization (supplementary Fig. 1). Cost types contributing most to the aggregated economic cost included the opportunity cost for health worker time (48 %), followed by the opportunity cost of non-health worker time (26 %) and capital costs for vehicles and equipment (19 %).

## Discussion

Sri Lanka operates a school-based, national HPV vaccination program that reaches Grade 6 girls. It has routinely reported reaching HPV vaccination coverage of about 90 % for eligible girls [16]. HPV vaccination sessions happen throughout the year with two peaks in February–March and September–October, reflecting the two-dose schedule with six months between doses. Most sessions provide fewer than 25 doses, likely reflecting the average class size for Grade 6.

The weighted mean financial cost per dose is low compared to what has been reported in the literature, with less than $0.30 spent in financial costs to deliver a dose of HPV vaccine [23]. The weighted mean economic cost per dose is closer to prior estimates at $3.88 per dose delivered [8], [9], [10], [11], [12], [13], [14].

The low financial costs incurred by the HPV vaccination program in Sri Lanka can be explained by several factors. Unlike many countries, in Sri Lanka no per diem or allowances (usually one of the main drivers of financial costs) are paid to the health workers. Also, HPV vaccination activities happen year-round and are fully integrated within the routine immunization and school health programs. Sri Lanka provides preventive and curative health care services under the integrated primary health care delivery system in Universal Health Coverage, which includes immunizations. Delivery of HPV vaccines through those channels enables economies of scale and scope with all other services provided. For example, all transportation is organized with existing vehicles owned by the Ministry of Health, preventing additional expenditures to rent vehicles or pay for public or private transportation. This level of integration into an existing school health program is likely a key success factor of the HPV vaccination program in Sri Lanka and may not be replicable in other settings where school health programs are not as developed. No outreach activities or immunization delivery in the community are organized, yet Sri Lanka sustains a high level of HPV vaccination coverage (99 % HPV1 and 82 % HPVc in 2019) [16]. This is likely due to a robust routine and integrated school-based delivery strategy. High coverage is another factor explaining the rather low financial cost to operate the program.

Additional cost efficiencies could be achieved by the HPV vaccination program in Sri Lanka. One way to further reduce the resources required to operate the program would be to organize larger vaccination sessions, although this may not be feasible as HPV vaccination session sizes currently depend on school sizes. Sri Lanka could also consider moving to a one-dose HPV vaccination schedule, as recent evidence has shown that this would offer solid protection and some countries have already made this change [3], [24], [25]. This would generate savings, primarily on vaccine procurement and supplies, which we have not included in our study, and could also reduce some of the costs related to service delivery.

Our study has several limitations. We collected data on the ongoing cost of the HPV vaccination program for the year 2019. The costs we report may not be reflective of costs for other years, although we consider them a good indication of the routine cost to operate HPV vaccination activities in the country. As a result of the COVID-19 pandemic and the ensuing economic and political crisis that occurred in Sri Lanka, we were only able to start collecting primary data in June 2022. We acknowledge that a recall bias may affect our result, but we tried as much as possible to minimize this by probing responses with documentation whenever available.

In addition, we are not reporting any cost for waste management as this is done at local hospitals and does not generate any cost at divisional health units. As local hospitals are not involved in HPV vaccination program activities, they were not included in our sample; hence, we did not collect any data from these locations and acknowledge this limitation. Although we are not able to assess waste management costs related to the HPV vaccination program, we believe that those costs would have a negligible effect on the economic costs, as waste related to HPV vaccines is disposed of with hospital waste, the latter likely representing much higher volumes.

At the district and national levels, we collected vehicle and human resources costs in a way that did not allow further disaggregation by program activity. Because of this, we report all cost of vehicles as part of vaccine collection or distribution and storage activities despite some vehicles being used for supervision activities. Additionally, all cost of health workers’ time is reported as part of a separate human resource category, even though this opportunity cost contributed to many program activities. At the district and national levels, costs related to estimating demand, vaccine procurement, program planning and management, social mobilization and IEC, training, and supervision are therefore underestimated.

Finally, we used the official World Bank currency exchange rate of 178.74 Sri Lankan rupees to US$1 to convert costs to 2019 current US$. Interpretation of results should be done with caution as the Sri Lankan rupee has been consistently depreciating against the US$ with a fast-growing trend in the last year. In 2021, the official exchange rate reported by the World Bank was 198.76 rupees to US$1 [21]. In 2023, the exchange rate increased to more than 300 rupees to the dollar [21].

This study provides key evidence to the government. It sheds light on the program implementation cost, which is critical to inform future decisions on any other new vaccine introduction with delivery organized through the school health program. Knowing the cost to deliver HPV vaccine is also key to inform a potential change to a one dose HPV vaccine schedule, which the country is currently exploring. Finally, the study findings are important to justify the continuation of fund allocations to the program in a financially difficult period for Sri Lanka.

## Conclusion

Sri Lanka operates a school-based HPV vaccination program that achieves high coverage and is characterized by low financial costs, which can inspire other countries to consider such integrated platforms. Sri Lanka's low financial costs may be due to the integrated school-based delivery strategy, which allows for economies of scope in a routinized program. Further, the high number of doses delivered per facility also enabled economies of scale and a low cost per dose. Our findings can inform ongoing financial and program planning in Sri Lanka, as well as provide insights to other LMICs considering the introduction of HPV vaccination using a school-based delivery strategy.

## CRediT authorship contribution statement

**Frédéric Debellut:** Writing – original draft, Visualization, Validation, Supervision, Software, Methodology, Investigation, Formal analysis, Conceptualization. **Deepa Gamage:** Writing – review & editing, Validation, Supervision, Project administration, Investigation. **Sandeep Kumar:** Writing – review & editing, Supervision, Project administration, Methodology, Conceptualization. **Sashimali Wickramasinghe:** Writing – review & editing, Validation, Supervision, Project administration, Investigation, Data curation. **Thilanga Ruwanpathirana:** Writing – review & editing, Validation, Supervision, Resources, Project administration, Investigation, Data curation. **Manjula Kariyawasam:** Writing – review & editing, Validation, Supervision, Project administration, Investigation, Data curation. **Chinthana Sanjeewa Perera:** Writing – review & editing, Validation, Supervision, Investigation, Data curation. **Samitha Ginige:** Writing – review & editing, Validation, Supervision, Project administration, Methodology, Investigation. **Nirmala Cooray:** Writing – review & editing, Validation, Supervision, Software, Resources. **Clint Pecenka:** Writing – review & editing, Project administration, Methodology, Funding acquisition. **Rose Slavkovsky:** Writing – review & editing, Software, Resources, Project administration, Methodology, Investigation, Funding acquisition, Formal analysis, Data curation, Conceptualization. **D. Scott LaMontagne:** Writing – review & editing, Validation, Supervision, Methodology, Funding acquisition, Formal analysis, Data curation, Conceptualization. **Mercy Mvundura:** Writing – review & editing, Validation, Supervision, Methodology, Funding acquisition, Formal analysis, Data curation, Conceptualization.

## Declaration of competing interest

The authors declare that they have no known competing financial interests or personal relationships that could have appeared to influence the work reported in this paper.

## Data Availability

Data will be made available on request.
