# Supplementary material for: Human papillomavirus (HPV) vaccination program in Sri Lanka: Ongoing costs and operational context of a routinized program
Source: Vaccine X. 2024 Feb 8;17:100456. doi: 10.1016/j.jvacx.2024.100456 (PMC10877402; doi:10.1016/j.jvacx.2024.100456)
Supplement: Supplementary data 1 [file mmc1.docx]

**Supplementary Table 1: Activities and related cost components included in the study**

| **Activity** | **Financial cost components** | **Opportunity cost components** |
| --- | --- | --- |
| Vaccine procurement | Amount paid for freight, clearance, insurance and taxes for HPV vaccines and supplies. | Value of health workers’ time spent on procuring HPV vaccine and supplies. |
| Estimating demand | None | Value of health workers’ time spent on estimating demand for HPV vaccine program. |
| Program planning and management | Per diem, travel allowances, venue hire, catering and other costs for management meetings. | Value of health workers and non-health workers’ time spent in microplanning and management meetings. |
| Social mobilization and IEC (information, education, and communication) | Per diem, travel allowances, venue hire, catering and other costs for social mobilization meeting and events.  Costs of producing and airing of TV and/or radio spots/other forms of media.  Printing and distribution of IEC materials. | Value of health workers and non-health workers’ time spent on social mobilization events. |
| Routine training | Per diem, travel allowances, venue hire, catering and other costs for training.  Production and printing  of training materials. | Value of health workers and non-health workers’ time spent in training. |
| Vaccine collection or distribution and storage | Per diems and travel allowances.  Cost of hired vehicles and public transport.  Fuel costs and vehicle maintenance.  Energy costs for cold storage. | Value of health workers’ time spent on vaccine collection or distribution and storage.  Annualized cost for vehicles, refrigerators, and vaccine carriers allocated for HPV vaccine. |
| Service delivery | Per diems and allowances for  vaccination teams to travel to vaccination sites (for outreach or school-based delivery).  Hired vehicles and public transport used to travel to vaccination locations.  Fuel costs and vehicle maintenance.  Expenditure for extra supplies done by HF/district. | Value of health workers and non-health workers’ time spent on vaccination activities.  Annualized cost for vehicles. |
| Supervision | Per diems and travel allowances.  Cost of hired vehicles and public transport. | Value of health workers and non-health workers’ time spent on supervision activities.  Annualized cost for vehicles. |
| Record keeping | Extra printing or copying costs for record keeping materials. | Value of health workers’ time spent on record keeping, reporting, monitoring and evaluation activities. |
| Waste management | Per diems and travel allowances.  Cost of hired vehicles and public transport.  Fuel for operating incinerators. | Value of health workers and non-health workers’ time spent on waste disposal.  Annualized cost of incinerators. |
| Crisis management | Per diems and travel allowances.  Other meeting costs or additional printing of IEC materials. | Value of health workers and non-health workers’ time spent on crisis management. |

**Supplementary Table 2: Secondary data (cost in 2019 US$).**

| **Human resources salaries** | |
| --- | --- |
| Medical officer (preliminary to grade 1) | $296 to $493 |
| Management positions (grade 3 to administrator grade) | $291 to $811 |
| Public health nursing sister (grade 1 to super grade) | $285 to $323 |
| Epidemiologist (grade 2 and grade 1) | $381 to $493 |
| Supervisory public health midwife / Public health midwife (grade 3 to super grade) | $381 to $338 |
| Public health inspector (grade 3 to grade 1) | $193 to $246 |
| Officer in charge (grade 3 to grade 1) | $181 to $355 |
| Health assistant | $157 |
| Development officer (grade 3 to grade 1) | $181 to $260 |
| School principal | $436 |
| Teacher | $261 |
| Support staff | $162 |
| Driver (grade 3 to grade 1) | $147 to $216 |
| Volunteer | $70 |
| **Equipment replacement prices** | |
| 4WD pickup truck | $38,324 |
| Three-wheeler | $4,168 |
| Refrigerator | $625 to $2,075 |
| Cold room | $16,307 to $21,224 |
| **Energy consumption and prices** | |
| Refrigerator energy consumption (kW per 24 hours) | 1.34 to 3.20 |
| Cold room energy consumption (kW per 24 hours) | 28 to 36 |
| Electricity (per kWh) | $0.036 |
| Diesel (liter) | $0.55 |
| Petrol (liter) | $0.77 |
| **HPV vaccine proportions** | |
| HPV proportion based on quantities at divisional health unit level | 0.0246 to 0.2583 |
| HPV proportion based on quantities at administrative level | 0.0378 to 0.0558 |
| HPV proportion based on volume at divisional health unit level | 0.1228 to 0.6526 |
| HPV proportion based on volume at administrative level | 0.1980 to 0.2705 |

**Supplementary Figure 1: Distribution of the aggregated economic cost per activity and cost type**
